# A Cross-Sectional Study of Extended-Spectrum *β*-Lactamase–Producing *Escherichia coli* Isolated From Clinical Samples: Single-Center Investigation in Indonesia

**DOI:** 10.1155/ijm/3743202

**Published:** 2025-08-21

**Authors:** Fitrotin Azizah, Dita Artanti, Yety Eka Sispita Sari, Anindita Riesti Retno Arimurti, Arya Iswara, Afifah Khairunnisa, Muhammad Evy Prastiyanto, Daniel Geleta

**Affiliations:** ^1^Diploma of Medical Laboratory Technology, Faculty of Health Science, Universitas Muhammadiyah Surabaya, Surabaya, Indonesia; ^2^Department of Medical Laboratory Technology, Faculty of Health and Nursing Science, Universitas Muhammadiyah Semarang, Semarang, Indonesia; ^3^Department of Medical Laboratory Sciences, Jimma University, Jimma, Oromia, Ethiopia

## Abstract

The emergence of extended-spectrum beta-lactamase–producing *Escherichia coli* (ESBL-producing *E. coli*) is a significant public health concern, particularly in developing countries like Indonesia, where reports on the prevalence and characteristics of these resistant strains are scarce. This lack of data hampers effective infection control and antibiotic stewardship efforts. This study is aimed at investigating the prevalence and assessing the antimicrobial susceptibility profiles of ESBL-producing *E. coli* isolated from clinical samples of Indonesian patients, thereby contributing to an understanding of antibiotic resistance patterns in this region. A cross-sectional study was conducted at RSUD dr. Adhyatma Hospital, Semarang, Indonesia, over 3 years (from January 2022 to December 2024). Clinical specimens were obtained from patients diagnosed with *E. coli* infections, and isolates were identified and assessed for antibiotic susceptibility utilizing the VITEK2 Compact system. Data were examined via the Fisher's exact test. Out of 449 *E. coli* isolates, 199 (44.3%) were identified as ESBL, with the highest prevalence in pus (35.6%) and urine (27.2%). ESBL-producing *E. coli* isolates demonstrated high sensitivity (above 90%) to amoxicillin–clavulanic acid, ceftazidime, ertapenem, meropenem, and tigecycline. Our study also underlined the higher prevalence of multidrug resistance (MDR) in ESBL compared to non-ESBL. The results highlight the urgent need for enhanced surveillance and infection control measures in healthcare settings to combat the spread of ESBL-producing *E. coli*. Healthcare professionals, including nurses and clinicians, must be aware of this resistance pattern to guide empirical treatment choices and improve patient outcomes in managing infections caused by these resistant strains.

## 1. Introduction

The emergence of antimicrobial resistance, especially from extended-spectrum *β*-lactamase–producing *Escherichia coli* (ESBL-producing *E. coli*), presents a substantial global health threat by often resulting in the ineffectiveness of empirical antibiotic treatment, hence, contributing to morbidity and mortality [1]. These bacteria are capable of hydrolyzing a wide range of beta-lactam antibiotics, which complicates treatment options for infections caused by them [2]. The increasing prevalence of ESBL-producing strains in various environments, including hospitals, community settings, and food sources, necessitates urgent attention to understanding their distribution and mechanisms of resistance [3].

The importance of addressing this problem lies in the rising rates of antibiotic resistance globally, which threaten effective infection management [4]. The prevalence of ESBL-producing *E. coli* in clinical samples from Indonesian patients is a significant public health concern. ESBLs are enzymes that confer resistance to a wide range of beta-lactam antibiotics, including penicillins and cephalosporins, making infections difficult to treat. This resistance leads to increased morbidity, mortality, and healthcare costs due to prolonged hospital stays and the necessity for more expensive alternative treatments [5, 6].

In Indonesia, the emergence of ESBL-producing bacteria is exacerbated by factors such as high antibiotic usage [7–9], inadequate infection control measures in healthcare settings, and the close interaction between humans and animals that can facilitate the spread of resistant strains [10, 11]. The situation is further complicated by the lack of comprehensive surveillance data on ESBL prevalence across different regions and populations within Indonesia, which hampers effective public health responses. Furthermore, questions remain regarding the factors contributing to ESBL-producing *E. coli* resistance patterns within Indonesian healthcare facilities. One main limitation of current studies is the lack of comprehensive data on the prevalence of ESBL-producing *E. coli* in clinical samples from Indonesian patients. The goal of the study is to fill this gap by providing detailed insights into the prevalence and antibiotic resistance profiles of ESBL-producing *E. coli* isolated from clinical samples in Indonesia, which may help inform public health interventions and antibiotic policies.

This research is aimed at answering critical questions regarding the prevalence rates of ESBL-producing *E. coli* in clinical samples and their resistance patterns. The hypothesis posits that there is a high prevalence of ESBL-producing strains among clinical isolates in Indonesia, significantly impacting treatment outcomes for patients. This cross-sectional study on ESBL-producing *E. coli* in Indonesia not only sheds light on the current state of antibiotic resistance but also serves as a foundation for future research and policy-making aimed at mitigating this growing public health threat. The findings will be instrumental in guiding interventions to improve patient outcomes and reduce the burden of resistant infections in Indonesia.

## 2. Methods

### 2.1. Research Design

This observational cross-sectional study was conducted in the Microbiology Laboratory of RSUD dr. Adhyatma Hospital in Semarang, Indonesia, over 3 years, from January 1, 2022, to December 31, 2024. This investigation encompassed all clinical specimens obtained at the Microbiology Laboratory, including samples from both inpatients and outpatients. The investigation encompassed clinical samples from individuals of various age demographics diagnosed with *E. coli* infections.

### 2.2. Population for Study and Criteria for Selection

The investigation encompassed all clinical specimens collected at the Microbiology Laboratory, comprising both inpatients and outpatients, amounting to a total of 2175 specimens. This study comprised *E. coli* isolates from 449 patients infected with *E. coli*. Each patient was assigned a single isolate of *E. coli*. This study utilized *E. coli* (both ESBL and non-ESBL) as the outcome variable, whereas age, gender, specimen type, and antibiotic susceptibility acted as predictor variables.

### 2.3. Collection of Specimens, Isolation, Identification, and Analysis of Antimicrobial Susceptibility Patterns

Retroactive data were collected from the microbiology laboratory documents, which were generated by microbiological operations in the following manner: The standard protocols were followed to collect the specimens, which were then inoculated onto MacConkey agar and incubated overnight at 37°C ± 2°C. Identification procedures were implemented in response to substantial bacterial proliferation in the specimen cultures. Initially, the bacteria were categorized based on the morphology of the colony, the margin, the elevation, the size, the shape, and the pigmentation. Using the VITEK2 Compact (bioMérieux, Craponne, France) device, all isolates were identified and their resistance patterns were evaluated [12–15].

Then, 16 antibiotics were evaluated against *E. coli*, comprising aminopenicillins (ampicillin (AM), ampicillin/sulbactam (AMS), and amoxicillin + clavulanic acid (AMC)); first-generation cephalosporin (cefazolin (CZO)); third-generation cephalosporins (ceftazidime (CAZ), cefotaxime (CTX), and ceftriaxone (CRO)); monobactam (aztreonam (AZM)); carbapenems (ertapenem (ETP) and meropenem (MEM)); aminoglycosides (gentamicin (GM)); fluoroquinolone (ciprofloxacin (CIP)); glycylcycline (tigecycline (TGC)); nitrofuran (nitrofurantoin (NIT)); and sulfonamides–trimethoprim (trimethoprim + sulfamethoxazole (SXT)). Multidrug-resistance (MDR) is defined as nonsusceptibility to ≥ 1 drug in ≥ 3 antimicrobial categories [16, 17].

### 2.4. Statistical Analysis

The data were analyzed using the Statistical Package for Social Sciences (SPSS, IBM, Version 27.0). The susceptibility of *E. coli* and other parameters was analyzed between ESBL and non-ESBL using Fisher's exact test. *p* values below 0.05 were considered significant.

## 3. Results

### 3.1. Demographic Characteristics

Of the 449 *E. coli* isolates, 202 (45%) were isolated from male patients and 247 (55%) from female patients. The highest isolation rate of *E. coli* was found in pus samples (35.6%), followed by urine samples (27.2%) and feces samples (23.4%) (Table 1).

### 3.2. Distribution of ESBL-Producing *E. coli* From Clinical Samples

Our data indicated a correlation between the ESBL-producing *E. coli* and diverse demographic and clinical specimens. A higher percentage of ESBL-producing *E. coli* was identified in females (59.8%) than in men (40.2%), with the peak prevalence occurring in the adult age group (25–64 years) at 60.3%. The isolation rate of ESBL-producing *E. coli* had no significant correlation with gender (*p* = 0.042) or age group (*p* = 0.136). However, significant results were shown in specimen type, with pus (45.7%) and urine (30.7%) being the most prevalent samples for ESBL-producing *E. coli* (Table 2).

### 3.3. Susceptibility Profiles of *E. coli*

**Figure 1:**
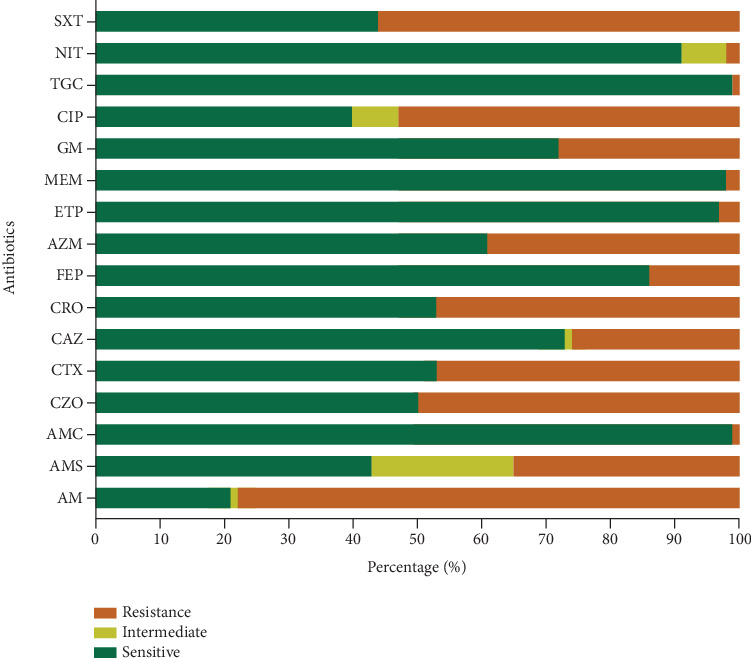
Susceptibility profiles of *E. coli* isolates. Aminopenicillins (AM: ampicillin; AMS: ampicillin/sulbactam; and AMC: amoxicillin + clavulanic acid); first-generation cephalosporin (CZO: cefazolin); third-generation cephalosporins (CAZ: ceftazidime; CTX: cefotaxime; and CRO: ceftriaxone); fourth-generation cephalosporin (FEP: cefepime); monobactam (AZM: aztreonam); carbapenems (ETP: ertapenem and MEM: meropenem); aminoglycosides (GM: gentamicin); fluoroquinolone (CIP: ciprofloxacin); glycylcycline (TGC: tigecycline); nitrofuran (NIT: nitrofurantoin); and sulfonamides–trimethoprim (SXT: trimethoprim + sulfamethoxazole).

The study of *E. coli* isolates showed significant differences in antibiotic susceptibility and resistance patterns between ESBL and non-ESBL strains. Among the 449 isolates analyzed, 250 (55.68%) were classified as non-ESBL and 199 (44.32%) as ESBL-producing *E. coli. E. coli* isolates showed significant sensitivity to AMC, ETP, MEM, TGC, and NIT (Figure 1).

### 3.4. Antibiotic Resistance Profile of ESBL-Producing *E. coli* and Non-ESBL

ESBL-producing *E. coli* exhibits a higher level of resistance to certain antibiotics. ESBL-producing *E. coli* was 100% resistant to AM, followed by 99% resistance to CZO, 98% resistance to CRO, 97% resistance to CTX, 82.9% resistance to AZM, and 86.4% resistance to CIP. AMC, CAZ, ETP, MEM, and TGC demonstrated high sensitivity rates above 90% (Table 3). MDR status with prevalence and frequency (percent) of resistant drugs is depicted in Table 4. The prevalence of MDR in ESBL-producing E. coli was high at 199 (100%), compared to 96 (38.4%) among non-ESBL strains, with a *p* value of < 0.001.

## 4. Discussion

The rise of multidrug-resistant *E. coli* strains, particularly those producing ESBLs, has been documented globally, yet comprehensive data from Indonesia remain scarce. Previous studies have indicated a significant prevalence of ESBL-producing *E. coli* in various regions, including Saudi Arabia and Ethiopia, but the specific epidemiology in Indonesia has not been thoroughly explored [18, 19]. This gap in research limits the ability to implement effective infection control and antibiotic stewardship strategies, particularly in healthcare settings where the burden of resistant infections is high [20].

In our study, 199 out of 449 *E. coli* isolates (44.3%) were identified as ESBL producers. The highest prevalence was in pus (35.6%) and urine (27.2%) samples. Notably, the ESBL-producing isolates demonstrated high sensitivity (over 90%) to antibiotics such as AMC, CAZ, ETP, MEM, and TGC. This finding aligns with previous research indicating that while ESBL-producing strains are often resistant to many antibiotics, they can still exhibit susceptibility to certain agents [21, 22]. Furthermore, our study revealed a higher prevalence of MDR in ESBL-producing isolates compared to non-ESBL strains, consistent with findings from other regions [22].

This finding aligns with previous research that has consistently reported high rates of ESBL-producing *E. coli* in clinical isolates, particularly in urinary tract infections (UTIs) and pus samples. For instance, a study conducted in Burkina Faso reported a similar prevalence of ESBL-producing *E. coli*, with 58% of isolates from urine samples being ESBL-positive [23]. Additionally, Mahdani et al. noted that ESBL-producing *E. coli* has emerged as a significant cause of UTIs in both community and hospital settings, corroborating our findings regarding the high prevalence in urine samples [24]. Similarly, this study is in line with a meta-analysis conducted in Egypt and highlights the high prevalence of ESBL-producing *E. coli* (64%) [25].

In terms of antibiotic susceptibility, our results indicated that ESBL-producing *E. coli* isolates exhibited high sensitivity (over 90%) to several antibiotics, including AMC, CAZ, ETP, MEM, and TGC. This sensitivity profile is consistent with findings from other studies. For instance, Abdel-Moaty et al. reported that all ESBL-producing *E. coli* isolates were susceptible to imipenem, suggesting that carbapenems remain effective against these resistant strains [26]. Similarly, a study by Akoachere et al. found that ESBL-producing *E. coli* isolates demonstrated high susceptibility rates to carbapenems, reinforcing the notion that these antibiotics are critical for treating infections caused by resistant *E. coli* [27].

Moreover, our study highlighted a higher prevalence of MDR among ESBL-producing *E. coli* compared to non-ESBL strains. This observation is supported by the work of Taneja et al., who noted that ESBL-producing *E. coli* isolates exhibited a significant resistance pattern, particularly against commonly used antibiotics [28]. The increased MDR in ESBL-producing strains is a growing concern in clinical microbiology, as it complicates treatment options and poses challenges for infection control.

While our study provides valuable insights, it is not without limitations. The cross-sectional design may not capture the full spectrum of resistance patterns over time, and the study was conducted at a single hospital, which may limit the generalizability of the findings to other regions in Indonesia. Additionally, the reliance on the VITEK2 Compact system for susceptibility testing, while reliable, may not account for all resistance mechanisms present in the isolates [29]. The lack of genotypic data (e.g., absence of ESBL and bla gene identification) limits molecular-level insight into resistance mechanisms. The absence of adjustment for confounding factors may limit causal inference. While Fisher's exact tests reveal associations, multivariate analysis would better account for possible confounders. Future studies should consider a multicenter approach and include molecular characterization of resistance genes to provide a more comprehensive understanding of the resistance landscape.

The findings of this study underscore the necessity for enhanced surveillance and research into the epidemiology of ESBL-producing *E. coli* in Indonesia. Future research should focus on identifying the sources of these resistant strains, including potential zoonotic transmission from livestock and food products, as highlighted in studies from Vietnam and other regions [30]. Additionally, exploring the impact of antibiotic usage patterns in both human and veterinary medicine on the emergence of resistance will be crucial [23].

## 5. Conclusion

In conclusion, the high prevalence of ESBL-producing *E. coli* in clinical samples from Indonesian patients highlights an urgent public health concern. The study's findings emphasize the need for improved infection control measures and antibiotic stewardship in healthcare settings. By raising awareness among healthcare professionals regarding the resistance patterns of *E. coli*, we can better guide empirical treatment choices and ultimately improve patient outcomes in managing infections caused by these resistant strains.

## Figures and Tables

**Table 1:** Demographic characteristics.

**Parameters**	**Inference**
*Escherichia coli* (%)	449 (100%)
Gender	
Male	202 (45%)
Female	247 (55%)
Types of samples, *n* (%)	
1. Pus	160 (35.6%)
2. Blood	11 (2.4%)
3. Body fluid	1 (0.2%)
4. Secret	31 (6.9%)
5. Urine	122 (27.2%)
6. Sputum	19 (4.2%)
7. Faeces	105 (23.4%)

**Table 2:** Correlation of *E. coli* (characterized as non-ESBL and ESBL) among research participants with demographic variables and other clinical specimens.

**Variable**	***E. coli***		**p** **value**
	**Non-ESBL**	**ESBL**	
Gender			
Male	122 (48.8%)	80 (40.2%)	0.042
Female	128 (51.2%)	119 (59.8%)	
Age groups (years)			
Children (0–14 years)	51 (20.4%)	31 (15.6%)	0.136
Youth (15–24 years)	22 (8.8%)	14 (7%)	
Adults (25–64 years)	151 (60.4%)	120 (60.3%)	
Seniors (65 years and over)	26 (10.4%)	34 (17.1%)	
Specimen type			
Pus	69 (27.6%)	91 (45.7%)	< 0.001⁣^∗^
Blood	4 (1.6%)	7 (3.5%)	
Body fluid	1 (0.4%)	0 (0%)	
Secret	25 (10%)	6 (3%)	
Urine	61 (24.4%)	61 (30.7%)	
Sputum	7 (2.8%)	12 (6%)	
Feces	83 (33.2%)	22 (11.1%)	

*Note*: ⁣^∗^*p* < 0.05 is considered significant.

**Table 3:** Antibiotic resistance profile of ESBL-producing *E. coli* and non-ESBL to different antimicrobial agents.

**Class**	**Antibiotics**	**Non-ESBL**	**ESBL**
Aminopenicillins	Ampicillin (AM)		
	Sensitive	92 (36.8%)	0
	Intermediate	3 (1.2%)	0
	Resistant	155 (62%)	199 (100%)
	Ampicillin/sulbactam (AMS)		
	Sensitive	125 (50%)	67 (33.7%)
	Intermediate	56 (22.4%)	43 (21.6%)
	Resistant	69 (27.6%)	89 (44.7%)
	Amoxicillin + clavulanic acid (AMC)		
	Sensitive	248 (99.2%)	198 (99.5%)
	Intermediate	0	0
	Resistant	2 (0.8%)	1 (0.5%)

First-generation cephalosporin	Cefazolin (CZO)		
	Sensitive	221 (88.4%)	2 (1%)
	Intermediate	2 (0.8%)	0
	Resistant	27 (10.8%)	197 (99%)

Third-generation cephalosporins	Cefotaxime (CTX)		
	Sensitive	231 (92.4%)	6 (3%)
	Intermediate	2 (0.8%)	0
	Resistant	17 (6.8%)	193 (97%)
	Ceftazidime (CAZ)		
	Sensitive	236 (94.4%)	92 (46.2%)
	Intermediate	1 (0.4%)	3 (1.5%)
	Resistant	13 (5.2%)	104 (52.3%)
	Ceftriaxone (CRO)		
	Sensitive	233 (93.2%)	4 (2%)
	Intermediate	2 (0.8%)	0
	Resistant	15 (6%)	195 (98%)

Fourth-generation cephalosporin	Cefepime (FEP)		
	Sensitive	242 (96.8%)	143 (71.9%)
	Intermediate	0 (0%)	1 (0.5%)
	Resistant	8 (3.2%)	55 (27.6%)

Monobactam	Aztreonam (AZM)		
	Sensitive	241 (96.4%)	33 (16.6%)
	Intermediate	0 (0%)	1 (0.5%)
	Resistant	9 (3.6%)	165 (82.9%)

Carbapenems	Ertapenem (ETP)		
	Sensitive	244 (97.6%)	193 (97%)
	Intermediate	1 (0.4%)	2 (1%)
	Resistant	5 (2%)	4 (2%)
	Meropenem (MEM)		
	Sensitive	245 (98%)	197 (99%)
	Intermediate	0 (0%)	0 (0%)
	Resistant	5 (2%)	2 (1%)

Aminoglycosides	Gentamicin (GM)		
	Sensitive	210 (84%)	112 (56.3%)
	Intermediate	1 (0.4%)	0 (0%)
	Resistant	39 (15.6%)	87 (437%)

Fluoroquinolone	Ciprofloxacin (CIP)		
	Sensitive	151 (60.4%)	19 (9.5%)
	Intermediate	21 (8.4%)	8 (4%)
	Resistant	78 (31.2%)	172 (86.4%)

Glycylcycline	Tigecycline (TGC)		
	Sensitive	249 (99.6%)	199 (100%)
	Intermediate	1 (0.4%)	0 (0%)
	Resistant	0 (0%)	0 (0%)

Nitrofuran	Nitrofurantoin (NIT)		
	Sensitive	231 (92.4%)	178 (89.4%)
	Intermediate	16 (6.4%)	16 (8%)
	Resistant	3 (1.2%)	5 (2.5%)

Sulfonamides–trimethoprim	Trimethoprim+ sulfamethoxazole (SXT)		
	Sensitive	136 (54.4%)	63 (31.7%)
	Intermediate	0 (0%)	0 (0%)
	Resistant	114 (45.6%)	136 (68.3%)

**Table 4:** Prevalence of multidrug resistance in ESBL-producing *E. coli* and non-ESBL.

**MDR status**	**Non-ESBL (** **n** = 250** )**	**ESBL (** **n** = 199** )**	**p** **value**
MDR	96 (38.4%)	199 (100%)	< 0.001⁣^∗^
Non-MDR	154 (61.6%)	0 (0%)	

*Note*: ⁣^∗^*p* < 0.05 is considered significant.

## Data Availability

The data that support the findings of this study are available on request from the corresponding author. The data are not publicly available due to privacy or ethical restrictions.
